# *In-situ* measurement of the heat transport in defect- engineered free-standing single-layer graphene

**DOI:** 10.1038/srep21823

**Published:** 2016-02-24

**Authors:** Haidong Wang, Kosaku Kurata, Takanobu Fukunaga, Hiroshi Takamatsu, Xing Zhang, Tatsuya Ikuta, Koji Takahashi, Takashi Nishiyama, Hiroki Ago, Yasuyuki Takata

**Affiliations:** 1 Department of Mechanical Engineering, Kyushu University, Fukuoka, 8190395, Japan; 2 Department of Engineering Mechanics, Tsinghua University, Beijing, 100084, China; 3 Department of Aeronautics and Astronautics, Kyushu University, Fukuoka, 8190395, Japan; 4 Institute for Materials Chemistry and Engineering, Kyushu University, Fukuoka, 8168580, Japan; 5 International Institute for Carbon-Neutral Energy Research, Kyushu University, Fukuoka, 8190395, Japan

## Abstract

Utilizing nanomachining technologies, it is possible to manipulate the heat transport in graphene by introducing different defects. However, due to the difficulty in suspending large-area single-layer graphene (SLG) and limited temperature sensitivity of the present probing methods, the correlation between the defects and thermal conductivity of SLG is still unclear. In this work, we developed a new method for fabricating micro-sized suspended SLG. Subsequently, a focused ion beam (FIB) was used to create nanohole defects in SLG and tune the heat transport. The thermal conductivity of the same SLG before and after FIB radiation was measured using a novel T-type sensor method on site in a dual-beam system. The nanohole defects decreased the thermal conductivity by about 42%. It was found that the smaller width and edge scrolling also had significant restriction on the thermal conductivity of SLG. Based on the calculation results through a lattice dynamics theory, the increase of edge roughness and stronger scattering on long-wavelength acoustic phonons are the main reasons for the reduction in thermal conductivity. This work provides reliable data for understanding the heat transport in a defective SLG membrane, which could help on the future design of graphene-based electrothermal devices.

Being a widely studied two-dimensional (2D) material, graphene has attracted increasing attention in a broad range of scientific fields^1^. Because of its ultra-strong *sp*^2^ bonding force between two neighboring carbon atoms, graphene has shown a remarkably high thermal conductivity^2^, *λ*, which makes it one of the most promising materials for spreading waste heat generated in micro or nanoelectronic devices^3^. However, the 2D structure of graphene is not robust and stable in nature^4^. Edge folding, scrolling, rippling or structural defects are readily generated during the graphene’s growth and processing. These defects will decrease the intrinsic thermal conductivity and lead to a deterioration in the performance of graphene-based devices^5^. On the other hand, it provides an opportunity to manipulate the heat transport in graphene by introducing different kinds of defects. This technique is rather useful in designing functional graphene devices. Within all these applications, the most fundamental problem is to achieve the quantitative correlation between the defects and thermal conductivity of graphene.

Recently, the Raman thermometry method has been used to measure the thermal conductivity of suspended single-layer graphene (SLG)^6^,^7^,^8^. The reported *λ* value is in a range of 1800 ∼ 5300 Wm^−1^ K^−1^. Because of the limited temperature sensitivity of Raman frequency shift, it is rather difficult to accurately measure the difference in the thermal conductivity of graphene caused by nano-scale defects. Alternatively, electrical methods could provide higher measurement accuracy and the temperature sensitivity is independent of the sample’s defect. The thermal conductivities of supported graphene and suspended graphene with different layers have been measured by using a microresistance thermometer^9^,^10^,^11^ method and modified T-type sensor^12^ method. At present, the reported data measured by electrical methods are still quite few because the fabrication process of microresistance device connected with suspended graphene is much more complicated than the Raman method.

In the present study, we report thermal conductivities of three types of suspended SLGs measured electrically by an *in-situ* T-type sensor method: (a) complete and flat graphene; (b) graphene with nanoholes induced by a focused ion beam (FIB) radiation; (c) graphene with edge scrolling. The highest *λ* value of type (a) graphene is found to be about 2300 Wm^−1^ K^−1^ near room temperature considering the contact thermal resistance. The Raman spectrum and scanning electron microscope (SEM) image were used to test the quality of fabricated graphene sample. Although no obvious polymeric residues or impurities were found on the suspended graphene, nano-scale residues may still remain after the MEMS process. In the supplementary material, the quality of suspended graphene membrane is discussed in detail. In this context, *in-situ* measurement means that the experiment was performed in an electron beam (EB)/FIB dual-beam system and the data of the same SLG sample were collected uninterruptedly before and after FIB radiation. By comparing these data, the influence of defect on thermal conductivity could be precisely captured. *λ* values of type (b) and type (c) SLGs are reduced by about 42% and 38%, respectively, compared with the type (a) SLG. The underlying mechanisms are explained using the theory of lattice dynamics. Several phonon scattering mechanisms are discussed in detail.

## Results

We developed a new method for suspending SLG ribbon connected to a microsensor. The fabrication details are given in the method section and supplementary material. Instead of long time wet etching, we used only 20 s XeF_2_ gas etching to remove the Si beneath the sensor. The etching depth was about 5 μm. During the etching process, the graphene was fully protected by EB-resist and SiO_2_ layer on the upper and lower surfaces, formed as a sandwich structure. The Raman spectrum of graphene sample is given in the supplementary material. The graphene microribbon was created by EB-lithography and O_2_ plasma etching. The O_2_ plasma caused unavoidable defect to the edges of graphene ribbon, thus a small D-band peak was found afterwards. Similar phenomenon was observed elsewhere in SLG^13^. The intensity ratio between D-band and G-band is 0.2. Based on a quantitative Raman evaluation result, there is only one defect per 4 × 10^4^ C atoms^14^. This low defect level should have negligible effect on the heat conduction process. In this work, six graphene samples were prepared for measurement, denoted from SLG1 to SLG6. These samples have different sizes, edge scrolling or defect conditions. High-resolution SEM images were taken for each sample. Because of the sufficient etching depth beneath SLG, the contrast between SLG and background is much better than the sample supported on substrate. Even the wrinkle and scrolled edges of SLG are clearly seen. It is worth noting that these SEM images were taken after the thermal measurement, because the EB radiation may cause damage to the crystal structure of graphene and decrease its thermal conductivity.

**Figure 1:**
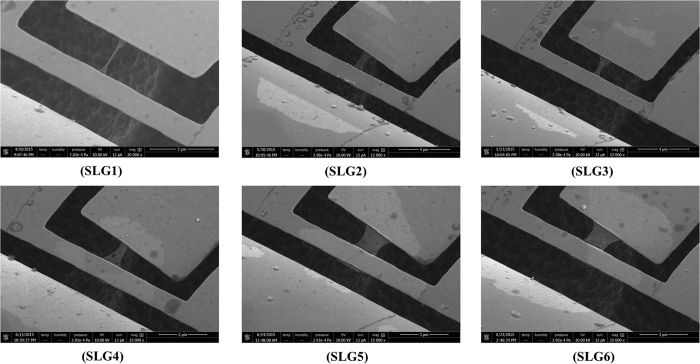
SEM images of SLG samples. SLG ribbon is connected to the middle of the Au nano-film sensor, named as “T-type” sensor method. The samples were tilted for a better observation. Six sensors have almost the same geometric dimensions. Six SLG samples were tested in the experiment: (SLG1) narrow sample with obvious edge scrolling from both sides to the center; (SLG2) narrow sample with noticeable edge scrolling; (SLG3) narrow sample with small edge scrolling; (SLG4) narrow sample with noticeable edge scrolling and two nanoholes; (SLG5 and SLG6) wide samples with small edge scrolling.

Figure 1 shows the SEM images of all six suspended SLG samples. SLG3, SLG5 and SLG6 are the complete and flat samples without any obvious defects. Therefore, their thermal conductivities are mostly close to the intrinsic properties of SLG. For the other samples, some edge or nanohole defects were randomly generated. In SLG1, an obvious edge scrolling is seen. The edges are scrolled from both sides to the center. In SLG4, two nanoholes are observed near the end connected to the sensor. Meanwhile, some small wrinkles are noticed in the SLG samples, indicating that the suspended SLG is not completely flat even without other defects. For SLG5 and SLG6, FIB radiation was employed to create nanoholes. Following an *in-situ* measurement procedure, the reduction in thermal conductivity was accurately measured.

The thermal conductivity of SLG was measured using a T-type sensor method, which was successfully applied to measure the individual carbon nanotubes^15^. The properties of suspended metallic nanofilm sensor were studied in detail in our previous work^16^,^17^. A brief principle of this method is given in the supplementary material. A commercial finite-element software COMSOL multiphysics was used for thermal analysis. As shown in the SEM image, the size of suspended Au sensor is comparable to the SLG sample, indicating a high temperature sensitivity. On the other hand, the suspended 100 nm thick Au sensor is very fragile during the measurement. Even small electrical current fluctuation or static electricity may break the sensor. The suspended Au sensor serves as a precise thermometer. Its thermal conductivity, temperature coefficient of resistance (TCR) may be slightly changed after overnight preservation or experiencing O_2_ plasma radiation. In order to avoid any possible influence to the measurement accuracy, the experiment was conducted according to the following steps: (1) the T-type sensor with SLG was placed on a Peltier heating/cooling stage in FEI Versa 3D EB/FIB dual-beam system. A four-wire method was used for resistance measurement; (2) the thermal conductance of Au sensor connected with SLG was measured under different temperature and electrical power conditions; (3) EB was turned on to take SEM images of SLG (see Fig. 1). After that, FIB was turned on for creating nanoholes or directly cutting SLG; (4) the thermal conductance of bare sensor was measured again under the same conditions as in step 2. The thermal conductivity of SLG can be calculated by comparing two thermal conductances measured in step 2 and step 4. This *in-situ* measurement procedure guarantees a high measurement accuracy to capture the effect of defects on thermal transport in graphene.

As described in the method section, the final step is to remove the protection layers of SLG. We carefully controlled the wet etching time for removing SiO_2_ beneath SLG (only 10s) and left about 40 nm thick SiO_2_ layer below the suspended Au sensor and SLG. The high-magnification SEM image of the cross-section of film sensor is given in the supplementary material. In this way, the contact area between SLG and Au film sensor (with 10 nm Cr adhesion layer) is in an Au-Cr/SLG/SiO_2_ sandwich structure. The reported interfacial thermal resistance for Au-Ti/graphene/SiO_2_ is 4 × 10^−8^ m^2^ KW^−1^ 18. A complete contact thermal resistance analysis is given in the supplementary material. Using a fin thermal resistance model^9^, the contact thermal resistance between graphene and thin film sensor contributes about 10% to the total thermal resistance of SLG.

**Figure 2:**
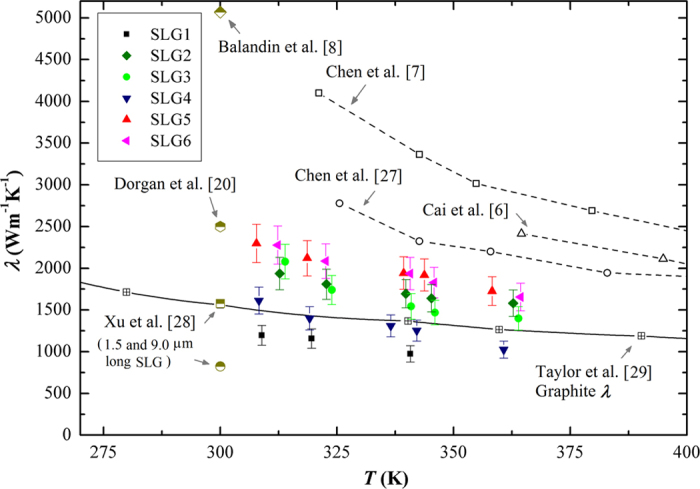
Measured thermal conductivities of SLG samples. The empty symbols are the experimental data from literatures. *λ* values measured by Raman method^6^,^7^,^8^,^27^ are all above 2000 Wm^−1^ K^−1^. Our experimental data (solid symbols) are close to the Raman measurement result.

Figure 2 shows the experimental results of all six SLG samples, comparing with the data from literatures. Table 1 shows the dimensions of SLG samples based on the SEM images. The largest SLG width at one end connected with Au sensor was used for calculation (see the supplementary material). The sample numbers (SLG1-SLG6) are ordered from small width to large width. The thickness of SLG is 0.334 nm as recommended in ref. 19. The temperature range of Peltier stage installed in the dual-beam system is from 263 K to 313 K. The temperature range in Fig. 2 is the result considering the average temperature rise of SLG during the measurement. It was reported that the defects and polymeric residues are the main reasons for the different *λ* values measured by Raman and electrical methods^9^. In our fabrication method, the SLG sample was protected by the resist and SiO_2_ layers during the gas etching processes. Defects and polymeric residues come from the last step of removing the protection layers. We optimized the wet etching parameters (time and temperature) to minimize the residues on SLG sample. However, nano-scale polymeric residues may still remain on the graphene surface. We discussed about the quality of graphene based on the Raman spectrum in the supplementary material. It is noted in Fig. 2 that the *λ* value extracted from the measured electric field data in ref. 20 is 2500 Wm^−1^ K^−1^ at room temperature, which is at the same level of our data. In this work, the SLG sample was carefully suspended and cleaned after removing the SiO_2_ layer beneath SLG. The authors also found that the clean samples mostly approached the intrinsic properties of graphene.

Except for SLG5 and SLG6, *λ* values of the other samples are relatively smaller because of the following reasons: smaller width, edge scrolling and nanohole defect. Comparing with SLG5 and SLG6, SLG2 and SLG3 have smaller widths. *λ* is decreased by about 15%. SLG4 has smaller width and two nanoholes. *λ* is decreased by about 36%. SLG1 has smaller width and obvious edge scrolling. *λ* is decreased by about 43%. More detailed analysis is given in the following sections. Considering the uncertainties of sensor calibration, finite-element analysis, temperature fluctuation of stage and size measurement, the total uncertainty of *λ* is estimated to be about 10%.

Perfect material properties of graphene come from its unique 2D crystal structure. On the other hand, it is possible to manipulate the properties of graphene by designing and changing its 2D structure. EB/FIB dual-beam system provides us a powerful tool for doing such nano-machining on graphene. In the experiment, we focused Ga^+^ ions in a shape of 100 nm line on SLG5 and SLG6. Under the condition of lowest current 1.6 pA and 30 kV accelerating voltage, the exposure dose is estimated to be about 2.3 × 10^−3^ pA/nm^2^. The thermal conductance of sensor was measured three times after 1s, 3s FIB radiation and complete cut-off of SLG.

**Figure 3:**
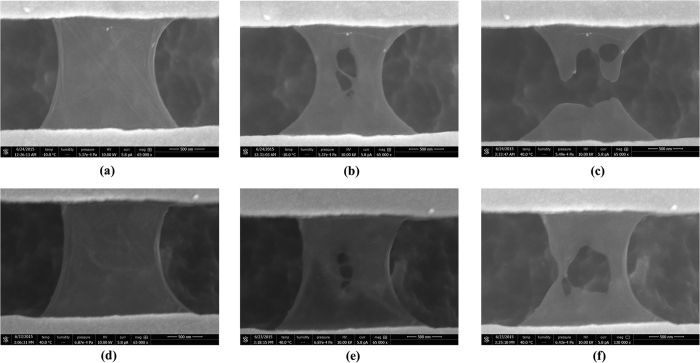
Nanoholes on SLG after FIB radiation. Nanoholes are easily created on SLG by short time FIB radiation. (**a**) SLG5 before FIB radiation; (**b**) SLG5 after 1s radiation; (**c**) SLG5 cut-off by FIB; (**d**) SLG6 before FIB radiation; (**e**) SLG6 after 1s radiation; (**f**) SLG6 after 3s radiation. SLG appears to be very fragile under FIB radiation. Even lowest current FIB could easily damage SLG. Another interesting phenomenon is that SLG ‘shrinks’ after FIB radiation. Its width at half-length decreases as the radiation time increases.

Figure 3 shows the SEM images of SLG samples after FIB radiation. It was observed that SLG was very fragile to FIB. Nanoholes were created after 1s radiation. The designed radiation area was 100 nm line in the middle of SLG. However, several nanoholes appeared at the same time and the size was much bigger than 100 nm. Meanwhile, the width of SLG at half-length decreases as the radiation time increases. This phenomenon may be related to the special mechanical characteristics of suspended SLG. An effective width was defined for the SLG with nanoholes in the thermal analysis (see the supplementary material). After 1s FIB radiation, the thermal conductivity of SLG5 is decreased to about 50% of the original value, even smaller than the value of the narrow SLG4 with naturally generated nanoholes. After 3s FIB radiation, the thermal conductivity is further decreased to about 20%.

## Discussion

In the framework of lattice dynamics, the thermal conductivity of SLG can be calculated as^21^:


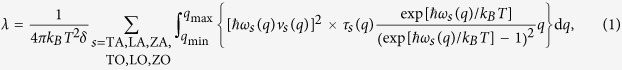


where *k*_*B*_, 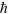, *ω*_*s*_, *τ*_*s*_, *q* and *T* are the Boltzmann constant, reduced Planck constant, phonon frequency, relaxation time, wave vector and temperature, respectively. *δ* = 0.35 nm is the interplanar spacing of graphite. *v*_*s*_ = d*ω*_*s*_/d*q* is the group velocity. The subscript *s* stands for six different phonon polarization branches, including three acoustic branches (TA, LA, ZA) and three optical branches (TO, LO, ZO). *ω*_*s*_, *v*_*s*_ and *τ*_*s*_ are mode-dependent variables, which are determined for each phonon wave vector and phonon branch. A full phonon dispersion relation from a valence-force field method is used for calculating *λ.* All six phonon branches have been taken into account^22^.

The relaxation time *τ*_*s*_ is determined by the following equation:


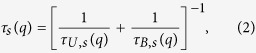


where *τ*_*U,s*_ and *τ*_*B,s*_ are the relaxation times of phonon Umklapp scattering and boundary scattering. These two relaxation times are given as^22^:


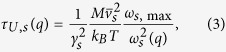



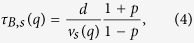


where *γ*_*s*_, 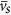
and *M* are the Gruneisen parameter, average phonon velocity and mass of a graphene unit cell, respectively. *ω*_s,max_ = *ω*_s_ (*q*_max_) is the maximum cut-off frequency. *d* is the width of graphene ribbon. *p* is a specularity parameter describing the roughness at the graphene edges.

**Figure 4:**
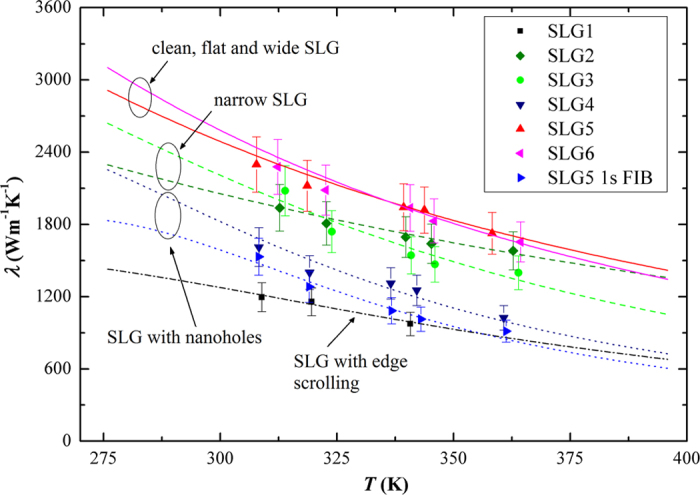
Comparison between the experimental data and theoretical calculation results. All the SLG samples are divided into four groups: (1) SLG5 and SLG6, the complete, flat and wide SLGs. *λ* is in a range of 1655 ∼ 2300 Wm^−1^ K^−1^; (2) SLG2 and SLG3, SLGs with smaller widths than the ones in the 1st group. *λ* is in a range of 1400 ∼ 1937 Wm^−1^ K^−1^; (3) SLG4 and SLG5 after 1s FIB radiation, SLGs with several nanoholes. *λ* is in a range of 913 ∼ 1611 Wm^−1^ K^−1^; (4) SLG1, edges scrolled from both sides to the center. *λ* is in a range of 973 ∼ 1195 Wm^−1^ K^−1^. The solid, dashed and dotted lines are the calculation results through equation (1).

Figure 4 compares all the measured *λ* values of SLGs (six samples and SLG5 after 1 s FIB radiation) and the theoretical calculation results through equation (1). In the calculation, *γ*_LA_ and *γ*_TA_ are chosen to be 1.80 and 0.75, respectively, as recommended in Ref. 21. It is found that the phonons of LA and TA modes are the dominant heat carriers for SLG. The ZA branch is also found to have non-negligible contribution. In the latest first-principles calculation, ZA branch phonons are found to have important contribution to the thermal transport in graphene^23^,^24^. Regarding the phonon scattering, both Umklapp and boundary scatterings are important for decreasing *λ* of SLG. For Umklapp scattering, the minimum cut-off frequency *ω*_s,min_ = *ω*_s_ (*q*_min_) plays an important role in determining *λ*. The acoustic phonons near the center of 1st Brillouin zone have long life time and high group velocity, contributing significantly to *λ*. Theoretically speaking, the one-atom thick SLG has no long-wavelength limit, because there is no coupling between the in-plane and cross-plane phonon modes^21^. However, the relaxation time of long-wavelength phonons tends to be infinite as *ω*_s,min_ approaches zero, which leads to an erroneous calculation result^25^. Based on our experimental results, *ω*_s,min_ is about 0.9 THz and 0.6 THz for LA and TA branches, respectively, which is much smaller than the value of 4 THz in bulk graphite^26^. This is a reflection of the 2D nature of heat transport in SLG. The long-wave length phonons have more important contribution to the heat transport in graphene than in bulk graphite. It is one of the reasons that the thermal conductivity of graphene is much higher than graphite 27,28,29 (see Fig. 2). Regarding the defect and size effects on *λ*, the following conclusions are obtained:

1. The width has a notable effect on *λ* when comparing the results of SLGs 5, 6 and SLGs 2, 3. Judging by their SEM images, there are no nanoholes or obvious edge scrolling. The specularity parameter *p* is between 0.85 and 0.90, indicating that the edge roughness is at the same level. Based on equation (4), the relaxation time *τ*_*B*_ is longer for wider SLGs, leading to a larger *λ*.
2. The defect of nanoholes decreases *λ* by about 42% when comparing the results of SLGs 4, 5-1s FIB and SLG6. It is noted that the width of SLG4 is almost the same as that of SLG5 after 1s FIB radiation. However, *λ* of SLG4 is higher. This finding indicates that the nanoholes created by FIB induce more limitation on *λ* than the naturally generated ones. This is reasonable because some additional damage to the lattice structure of graphene could be caused during FIB radiation. *λ* of SLG6 after 3s FIB radiation is further decreased to 470 Wm^−1^K^−1^.
3. The defect of edge scrolling decreases *λ* by about 38% when comparing the results of SLG1 and SLGs 2, 3. These SLGs have similar widths. The specularity parameter *p* of SLG1 is 0.53, which is much smaller than the value of SLG3, 0.85. Apparently, the scrolled edges are more “rough” and cause stronger phonon scattering. Furthermore, the scrolled graphene is no longer single layer. The interlayer phonon scattering will decrease the thermal conductivity of graphene. It is found that the minimum cut-off frequency *ω*_s,min_ is slightly higher for SLG1, indicating that more long-wavelength phonons, which are the main contributors to *λ*, are “cut off” because of the interlayer coupling.

In summary, we have developed a new method for preparing micro-sized suspended SLG. Thermal conductivities of six suspended SLG samples with different widths and defects were measured using a T-type sensor. FIB was employed to create nanoholes in the suspended SLG. Both edge scrolling and nanoholes have remarkable deterioration effect on *λ*. An increase of edge roughness and stronger scattering on long-wavelength acoustic phonons are believed to be the main reasons behind the experimental observations.

## Methods

In order to achieve micro-sized free-standing graphene sample for electrical measurement, we developed a new fabrication method. A SLG grown by chemical vapor deposition (CVD) method was transferred onto SiO_2_/Si substrate using a traditional PMMA method. Then the graphene layer was cut into micro ribbons by electron beam (EB) lithography and O_2_ plasma etching. After that, a pattern of T-type sensor was drawn by EB lithography on 300 nm thick EB-resist layer, which was spin-coated onto the graphene ribbon. 100 nm Au film was deposited by EB physical vapor deposition (PVD) method. After lift-off process in 45 °C ZDMAC solution, the T-type sensor was created and the graphene ribbon was located in the middle of the sensor. Subsequently, 600 nm thick EB-resist layer was spin-coated and patterned by EB lithography on the chip as a protection layer for graphene. After that, the SiO_2_ layer not covered by EB-resist was removed by 60 s reactive ion etching (RIE). The graphene ribbon was sandwiched between the EB-resist and SiO_2_ layer. Then the T-type sensor connected with graphene ribbon was suspended from the substrate after 20 s XeF_2_ gas etching. The etching depth was about 5 μm. Finally, the EB-resist and SiO_2_ layer were removed by ZDMAC solution and buffered hydrofluoric acid, followed by sufficient DI water rinse. A supercritical point dryer was used to release the device from water to avoid possible damage caused by the surface tension. It is worth noting that we carefully controlled the etching time to minimize the defects and chemical residues on graphene. Please refer to the supplementary material for more fabrication details.

## Additional Information

**How to cite this article**: Wang, H. *et al*. *In-situ* measurement of the heat transport in defect-engineered free-standing single-layer graphene. *Sci. Rep.* **6**, 21823; doi: 10.1038/srep21823 (2016).

## Supplementary Material

Supplementary Information

## Figures and Tables

**Table 1:** Dimensions of SLG samples.

Parameters	SLG1	SLG2	SLG3	SLG4	SLG5	SLG6
Length (μm)	1.86	1.56	1.65	1.62	1.62	1.73
Width (μm)	0.42	0.85	1.22	1.30	1.92	2.08
