# Supplementary material for: In-situ measurement of the heat transport in defect- engineered free-standing single-layer graphene
Source: Sci Rep. 2016 Feb 24;6:21823. doi: 10.1038/srep21823 (PMC4764828; doi:10.1038/srep21823)
Supplement: Supplementary Information [file srep21823-s1.doc]

**Supplementary material**

**In-situ measurement of the heat transport in defect-engineered**

**free-standing single-layer graphene**

Haidong WANG^1^, Kosaku KURATA^1^, Takanobu FUKUNAGA^1^, Hiroshi TAKAMATSU*^1^,

Xing ZHANG*^2^, Tatsuya IKUTA^3^, Koji TAKAHASHI^3^, Takashi NISHIYAMA^3^, Hiroki AGO^4^, Yasuyuki TAKATA^5^

^1^Department of Mechanical Engineering, Kyushu University

^2^ Department of Engineering Mechanics, Tsinghua University

^3^Department of Aeronautics and Astronautics, Kyushu University

^4^Institute for Materials and Chemical Research, Kyushu University

^5^International Institute for Carbon-Neutral Energy Research, Kyushu University

*Corresponding Author, E-mail: takamatsu@mech.kyushu-u.ac.jp, Fax: +81 92-802-3123

*Corresponding Author, E-mail: x-zhang@tsinghua.edu.cn, Fax: +86 10-62781610

**1. Fabrication process**

As described in the Method section of main text, the flow chart of fabrication process of single-layer graphene (SLG) sample is given in Fig. S1.


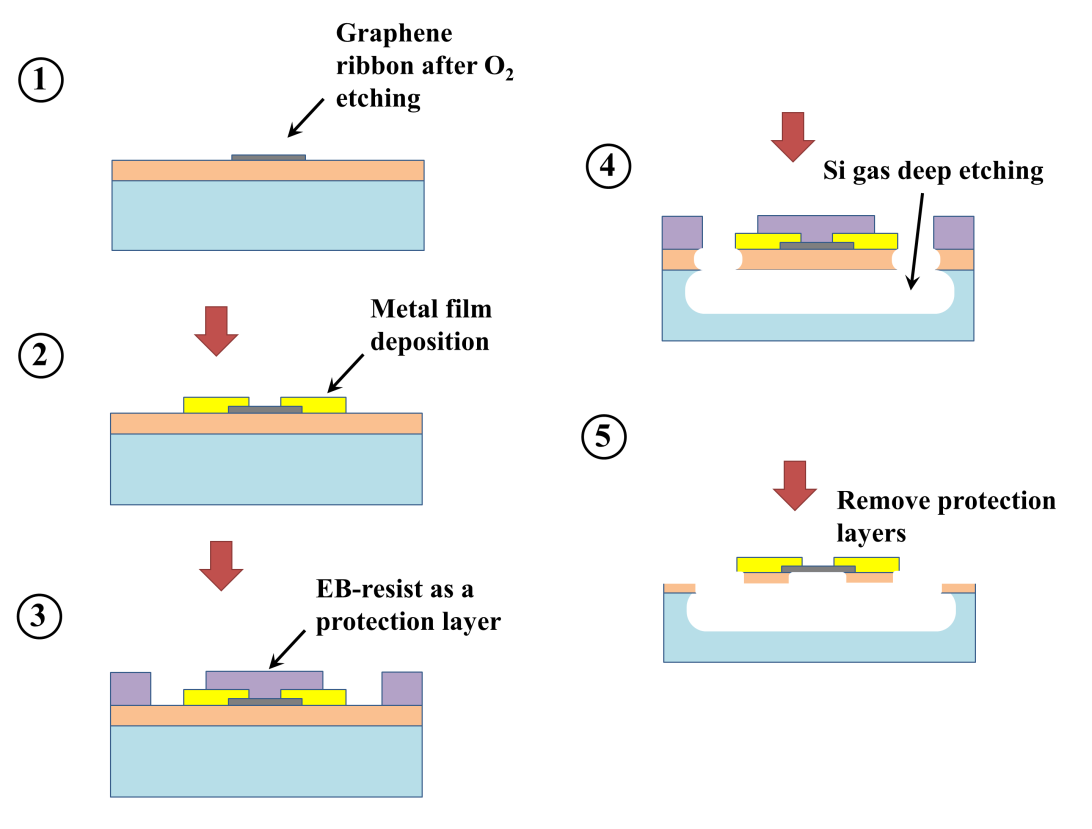


**Figure S1 Flow chart of fabrication process of suspended SLG.**

The SLG grown by chemical vapor deposition (CVD) method was transferred onto a SiO_2_/Si substrate and cut into micro ribbons using a standard electron beam (EB) lithography and O_2_ plasma etching. Then, 100 nm thick patterned Au film was deposited on top with 10 nm thick Cr adhesion layer. Subsequently, 600 nm thick EB-resist was spin-coated as a protection layer and the EB-resist/SLG/SiO_2_ sandwich structure was suspended after XeF_2_ gas etching process. Finally, the EB-resist and SiO_2_ layers were removed by ZDMAC solution and buffered hydrofluoric acid (BHA). The suspended SLG sample was dried using a supercritical point dryer to avoid any possible damage caused by the surface tension.

**2. Raman spectrum of SLG and sample quality**


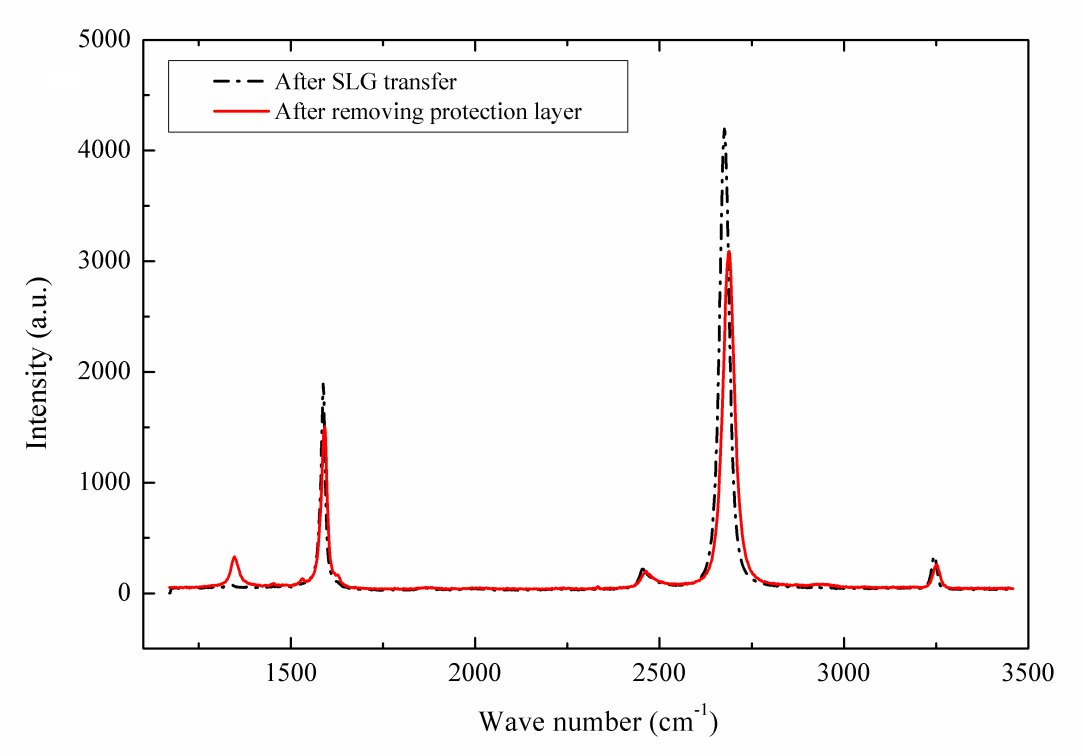


**Figure S2 Raman spectra of SLG after transfer and removal of protection layer.**

Figure S2 shows the Raman spectra of SLG measured after transfer to the substrate (black dash-dot line) and after removal of protection layer (red solid line). According to the Raman measurement in Ref. 1, the defect or polymer residue could lower the intensity of 2D-band with respect to the G-band. Although a small D-band peak is observed for the fabricated SLG, the base line noise is very small and the ratio between 2D-peak and G-peak is almost the same as the pristine graphene. These are important standards for judging the quality of graphene sample. In a quantitative Raman evaluation for the defect level of single-layer graphene, if the intensity ratio between D-band and G-band is 0.2 (same in our sample), there is only one defect per 4×10^4^ C atoms^2^. This low defect level should have negligible effect on the thermal transport in graphene.

In our experience, it is very easy to generate D-peak in the single-layer graphene even only by electron beam radiation in SEM. However, the situation is much different for the few-layer graphene. We do believe that the D-peak observed in our graphene sample is due to the O_2_ plasma etching for fabricating micro-ribbons, not the subsequent suspending process, because we measured the Raman spectrum of graphene ribbon supported on SiO_2_/Si substrate right after the etching process and found the same D-peak. Similar small D-peak was observed in the suspended graphene micro-ribbon fabricated by other groups^3^. In contrast, there is no D-peak observed in the few-layer graphene sample after the same O_2_ plasma treatment as stated in Ref. 4. The O_2_ plasma caused unavoidable defects at the edges of graphene ribbon, while the rest area was protected by the EB-resist layer. The size of focused laser beam in the Raman measurement was around 2 μm, covering the defective edges and the D-peak could be detected. If the focused laser spot is smaller than the width of graphene ribbon, this D-peak may not be observed.

**3. About the chemical residue on SLG**

It is reported that the polymeric residue is an important reason for the decreased thermal conductivity of graphene^1^. We tried several methods to remove the polymeric residues on the graphene sample. It is normally recommended to use a high temperature annealing process to remove the chemical residue. However, the thermal expansion of metallic film during the annealing process could cause cracks to the suspended SLG or even break it. Fig. S3 shows the scanning electron microscope (SEM) images of one SLG sample before and after the annealing process.


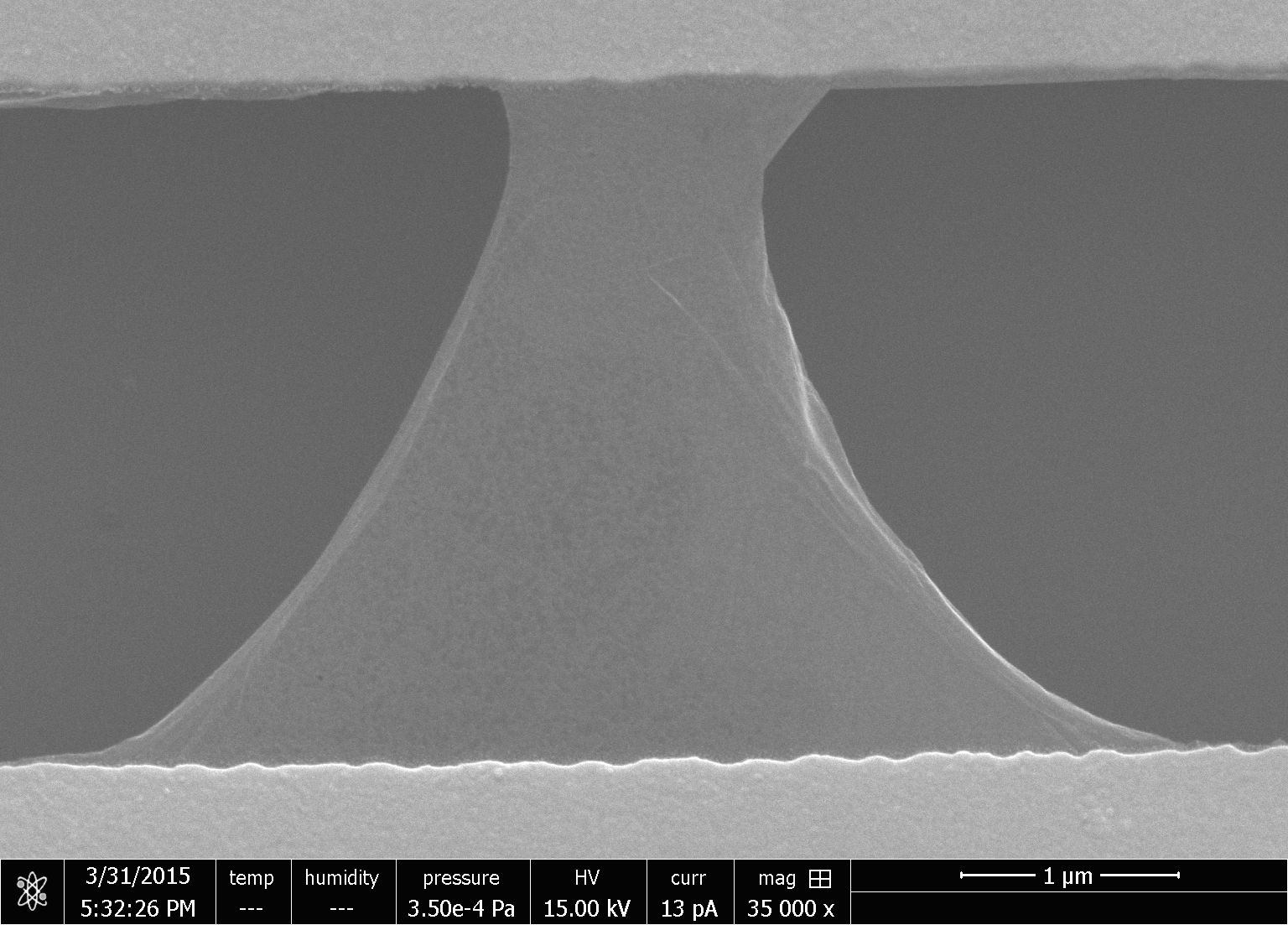

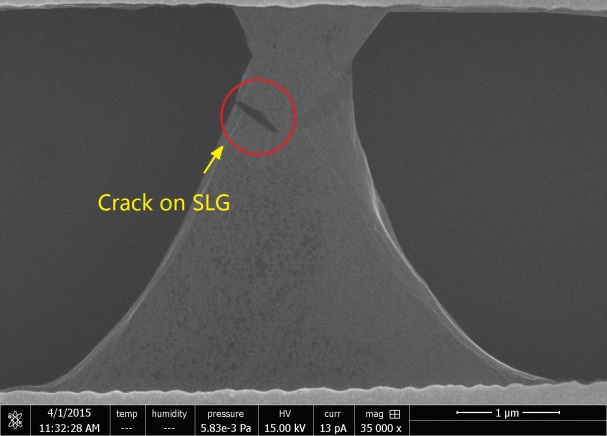


(a) (b)

**Figure S3 Crack on SLG caused by thermal expansion during annealing.** (a) suspended SLG sample before annealing; (b) suspended SLG with an obvious crack after annealing.

An obvious crack is seen on the suspended SLG after the annealing process. Instead of annealing, we found that the sufficient immersion time and temperature are important to remove the chemical residue and get clean graphene samples. These parameters vary for different polymeric layer and etching solution. In our case, 10 minutes in 45ºC ZDMAC solution is sufficient for removing the protection layer.


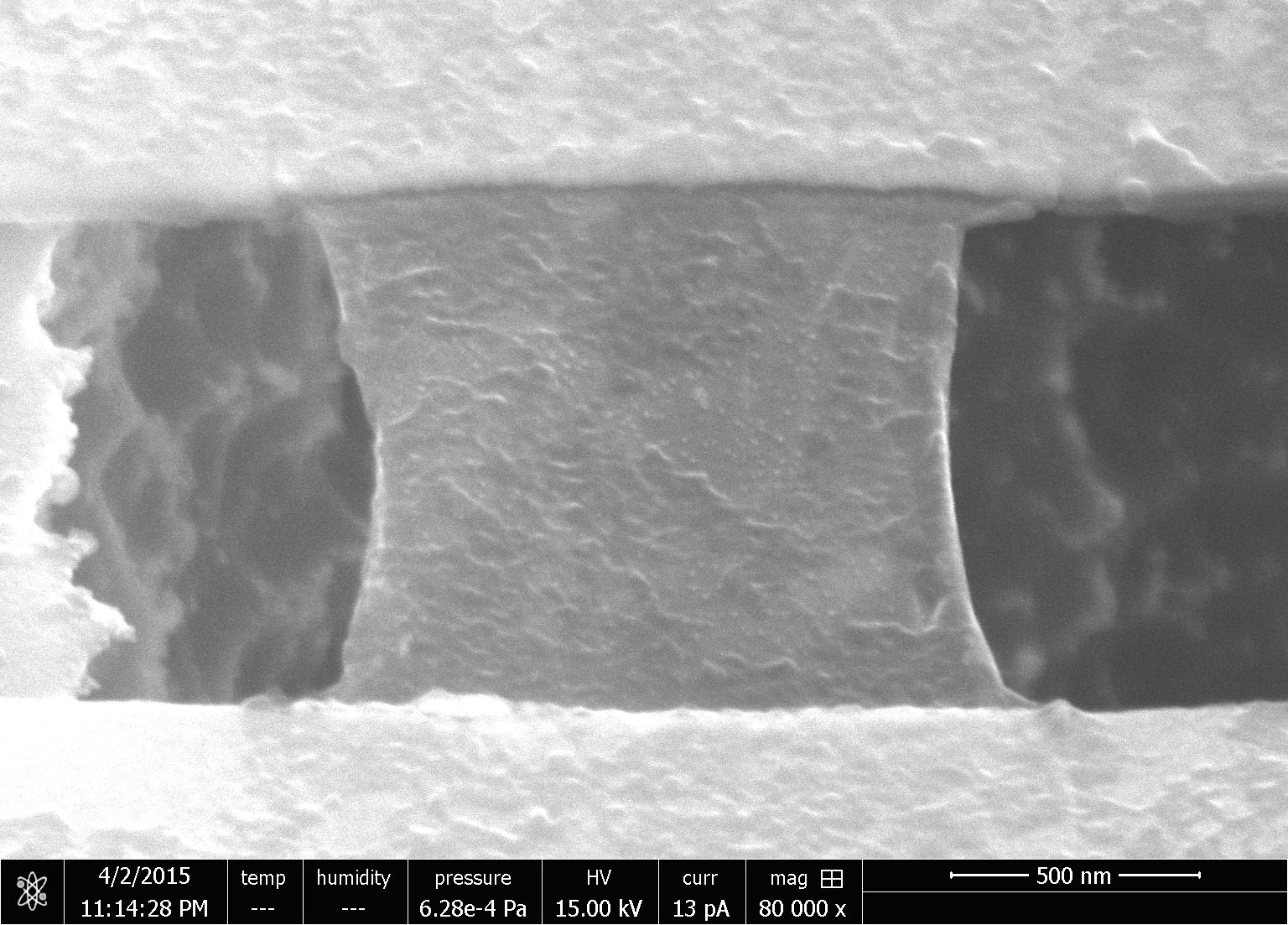

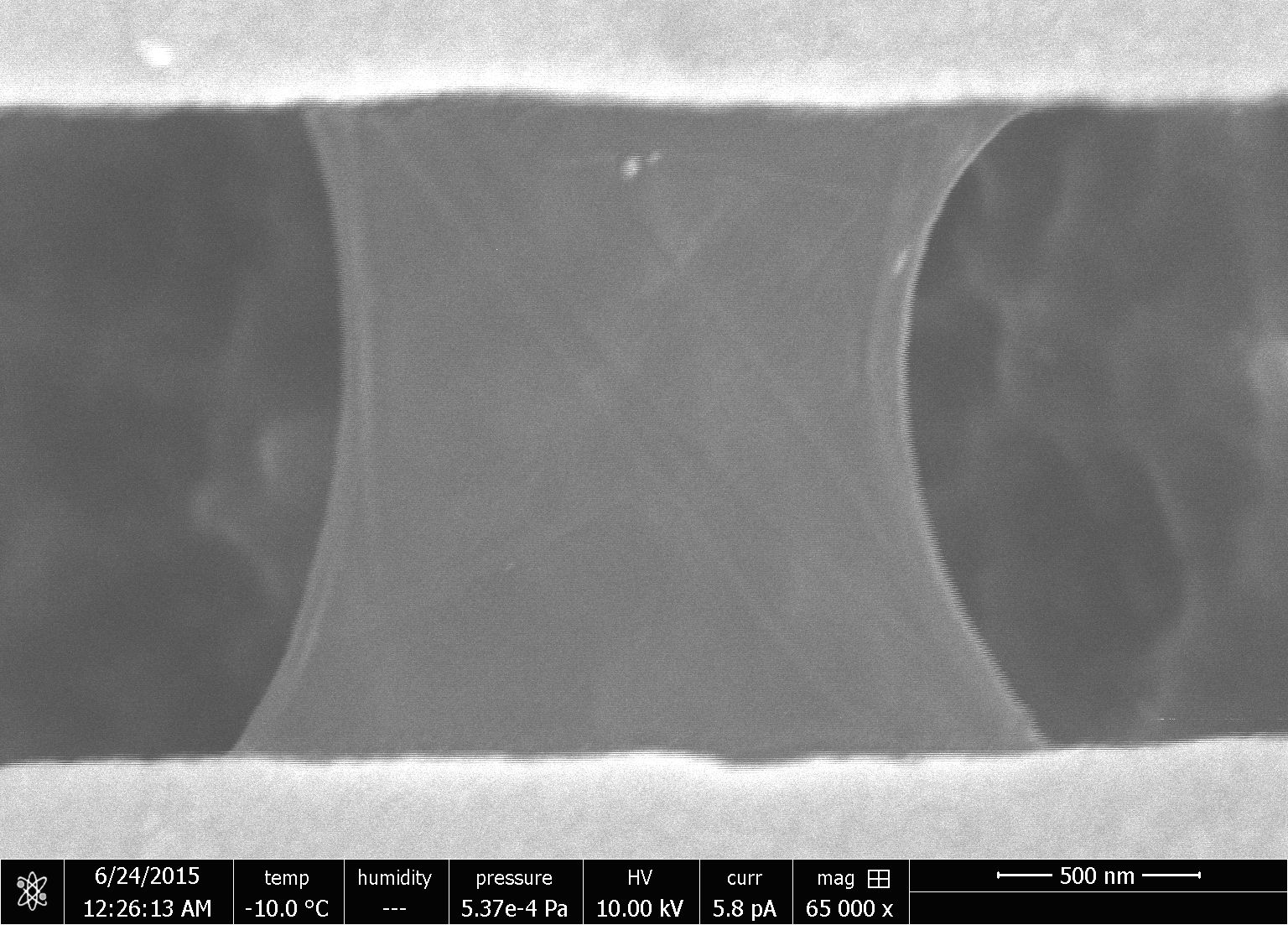


(a) (b)

**Figure S4 Comparison between the suspended SLG with and without polymeric residue layer.** (a) suspended SLG with a thin layer of polymeric residue; (b) suspended clean SLG used for thermal measurement in this work.

Figure S4 shows the SEM images of suspended SLG with and without polymeric residue layer. The immersion time of SLG sample in Fig. S4a was not enough and a thin layer of polymeric residue was left after drying. Differently, there is no polymeric residues observed in Fig. S4b. However, some nano-scale residues may be still left on graphene beyond the SEM resolution. Raman spectrum could provide some useful information about the chemical residues left on graphene. During the Raman measurement, the laser beam could be scattered by the residues and increase the background noise accordingly. Our graphene sample shows a flat baseline without any fluctuation. This could be an evidence for the low residue level of our sample. The D-peak in our sample was caused by O_2_ plasma etching and not related to the polymer residue generated in the subsequent suspending process.

**4. Determination of the width of SLG**


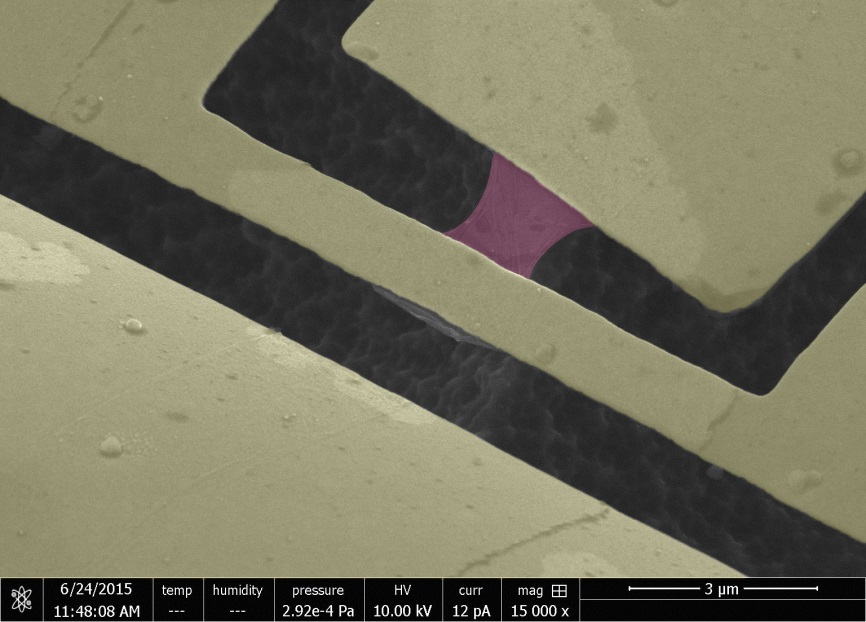


**Figure S5 SEM image of SLG5 shown with fake color.**


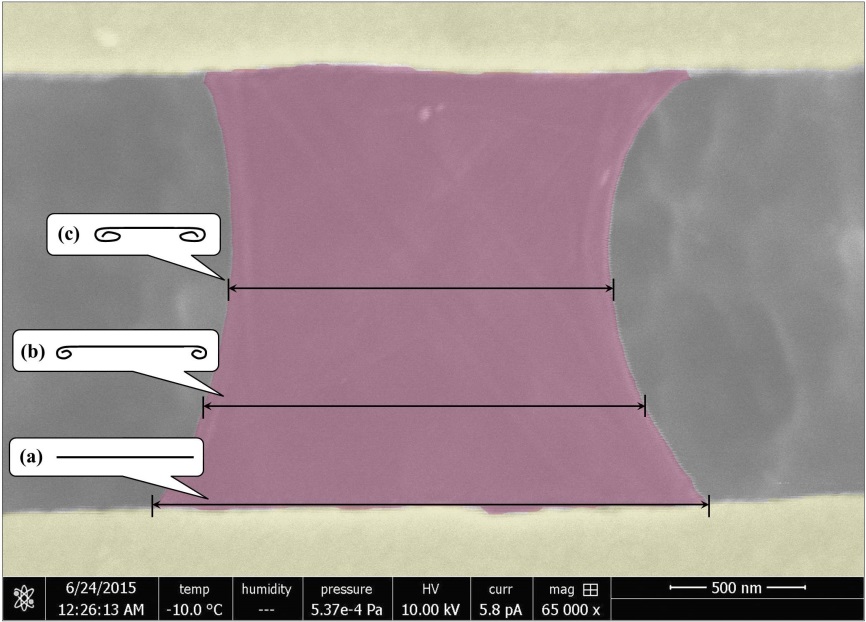


**Figure S6 SEM image of SLG5 under high magnification condition.** (a) largest width without edge scrolling; (b) small edge scrolling at the edge; (c) smallest width at half-length due to the edge scrolling.

Figures S5 and S6 show the SEM images of SLG5 with fake color. Because the SLG is clamped between the Au film and SiO_2_ layer at two ends, there is no edge scrolling at the end and the width is the largest (Fig. S5a). In the suspended part, edge scrolling is noticeable and decreases the width of SLG. Although the edge of SLG is scrolled, the cross-sectional area of SLG should be almost the same. From this point of view, we used the largest width at the end of SLG for calculation in this work.


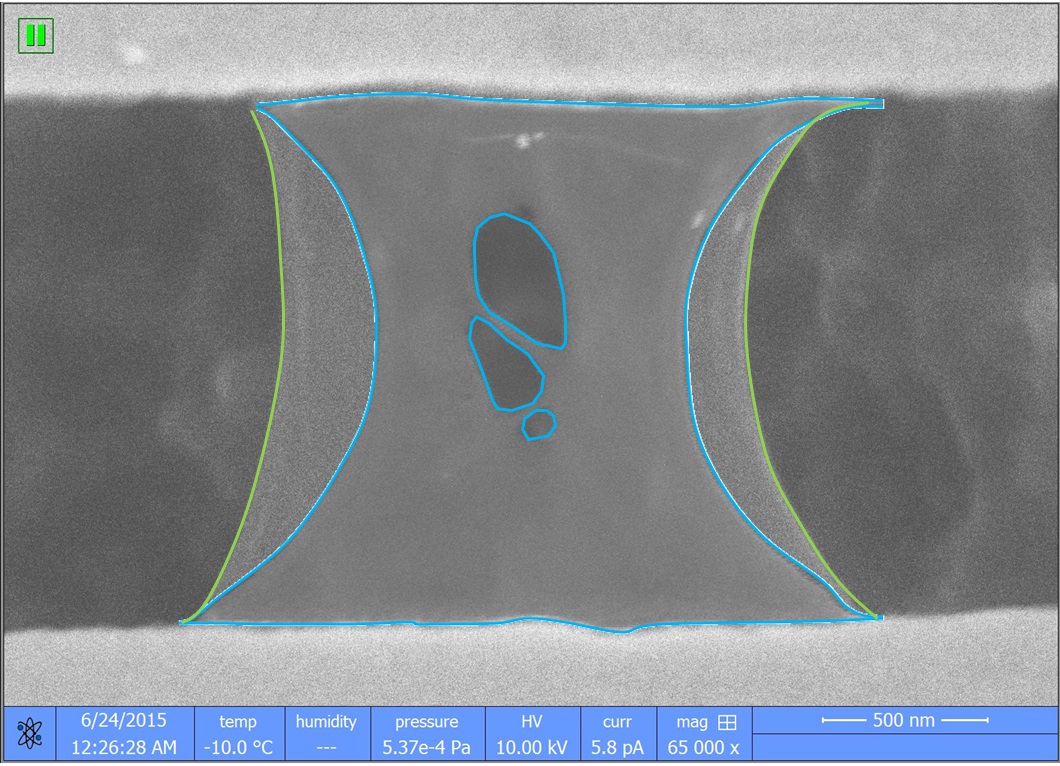


**Figure S7 Comparison between the width of SLG before and after FIB radiation.**

As shown in figure S7, the width of SLG decreased after FIB radiation. The blue and green curves marked the outlines of SLG with and without nanoholes. The effective width of SLG with nanoholes was calculated as its original width times an integral ratio between these two outlines. The hole-area was also subtracted from the total value. For example, the width of SLG5 was decreased from 1.92 μm to 1.32 μm after 1s FIB radiation. This effective width was used for calculating the thermal conductivity in this work.

**5. Measurement principle of T-type sensor**

T-type sensor method has been successfully applied for measuring the thermal conductivity of multi-walled carbon nanotube^5^. This method is simple and accurate for measuring the thermal conductivity of 1D and 2D nanomaterials.


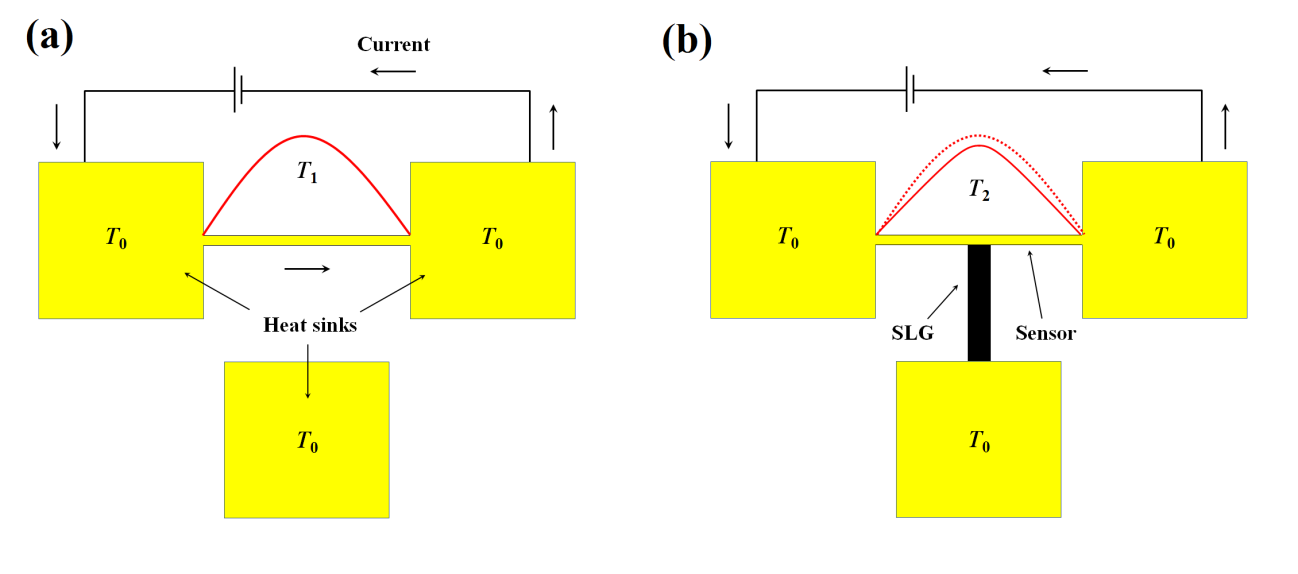


**Figure S8 Principle of T-type sensor method for measuring thermal conductivity.** (a) the bare nano-film sensor is heated by a direct current. *T*_1_ is the temperature distribution of the sensor; (b) a suspended SLG ribbon is connected to the sensor. The temperature distribution is decreased from *T*_1_ to *T*_2_.

Figure S8 shows the principle of the T-type sensor method. In vacuum, a suspended metallic sensor (100 nm thick Au film) is heated by a direct current and a parabolic temperature distribution *T*_1_ is established. Then, the same sensor connected with a suspended SLG ribbon is heated by the same electrical power. The SLG provides an additional heat conduction channel and the temperature distribution of sensor is decreased from *T*_1_ to *T*_2_. The metallic sensor is a precise resistance thermometer and the average temperatures $\bar{T}_{1}$ and $\bar{T}_{2}$ are measured respectively. The thermal conductivity of SLG can be calculated through the difference between $\bar{T}_{1}$ and $\bar{T}_{2}$. It is worth noting that the suspended nano-film sensor is very sensitive and its properties could be changed by O_2_ plasma treatment or other micro electro mechanical systems (MEMS) processes. In order to accurately capture the difference in thermal conductivity induced by nanodefects, we used an *in-situ* measurement procedure as described in the main text. The FIB was used to create nanoholes in SLG or cut off the sample.

We chose a commercial finite-element software COMSOL Multiphysics to calculate the thermal conductivities of all the samples. The measured temperature dependent thermal conductivity of Au film sensor was used as input parameters. Based on the calculation result, the temperature rise at the end of sensor is about 30 K at the maximum Joule heating power. A small temperature rise at the heat sink connected with graphene was also observed. The temperature distribution of sensor calculated by the finite-element model is shown in figure S9.


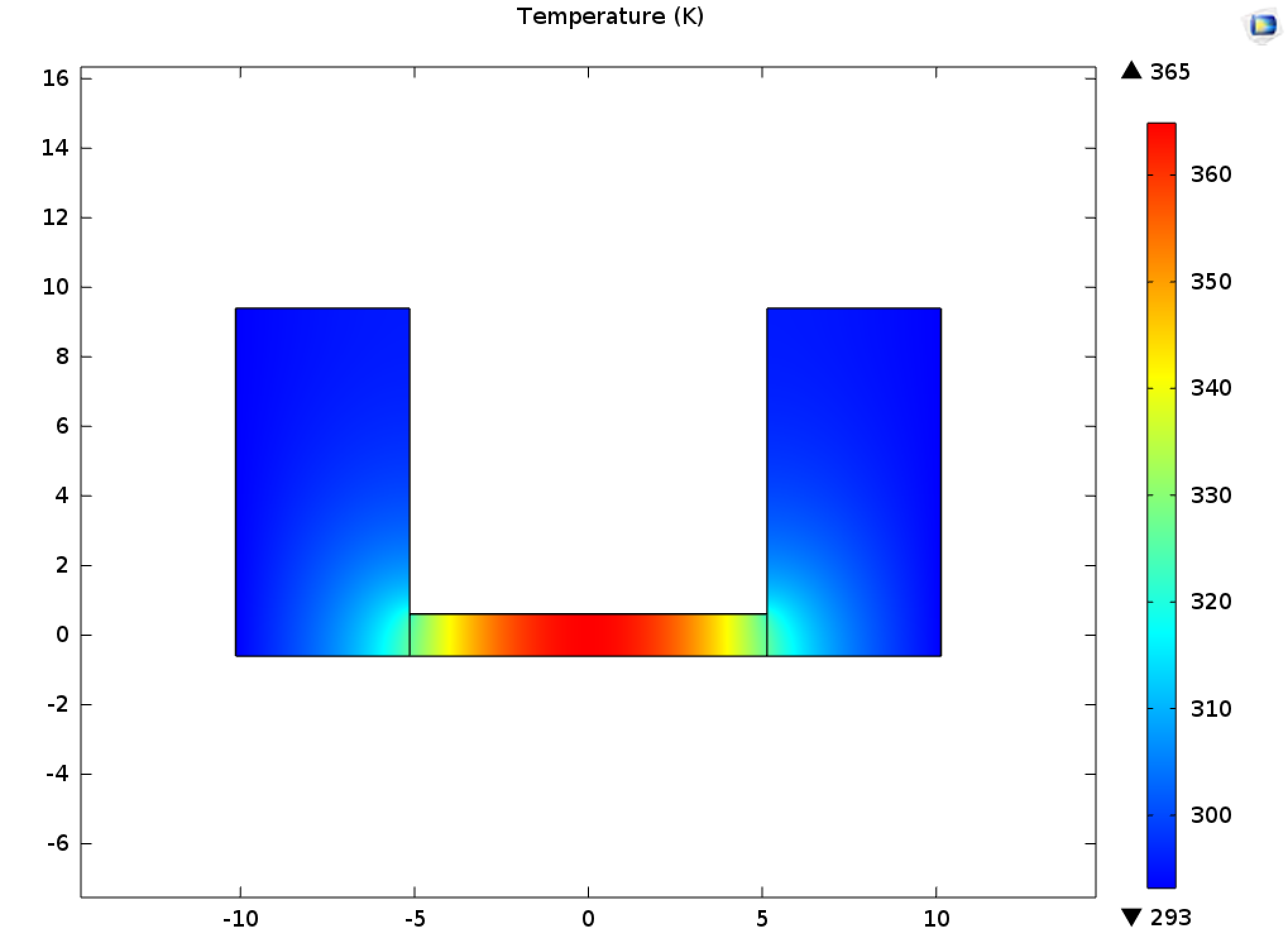

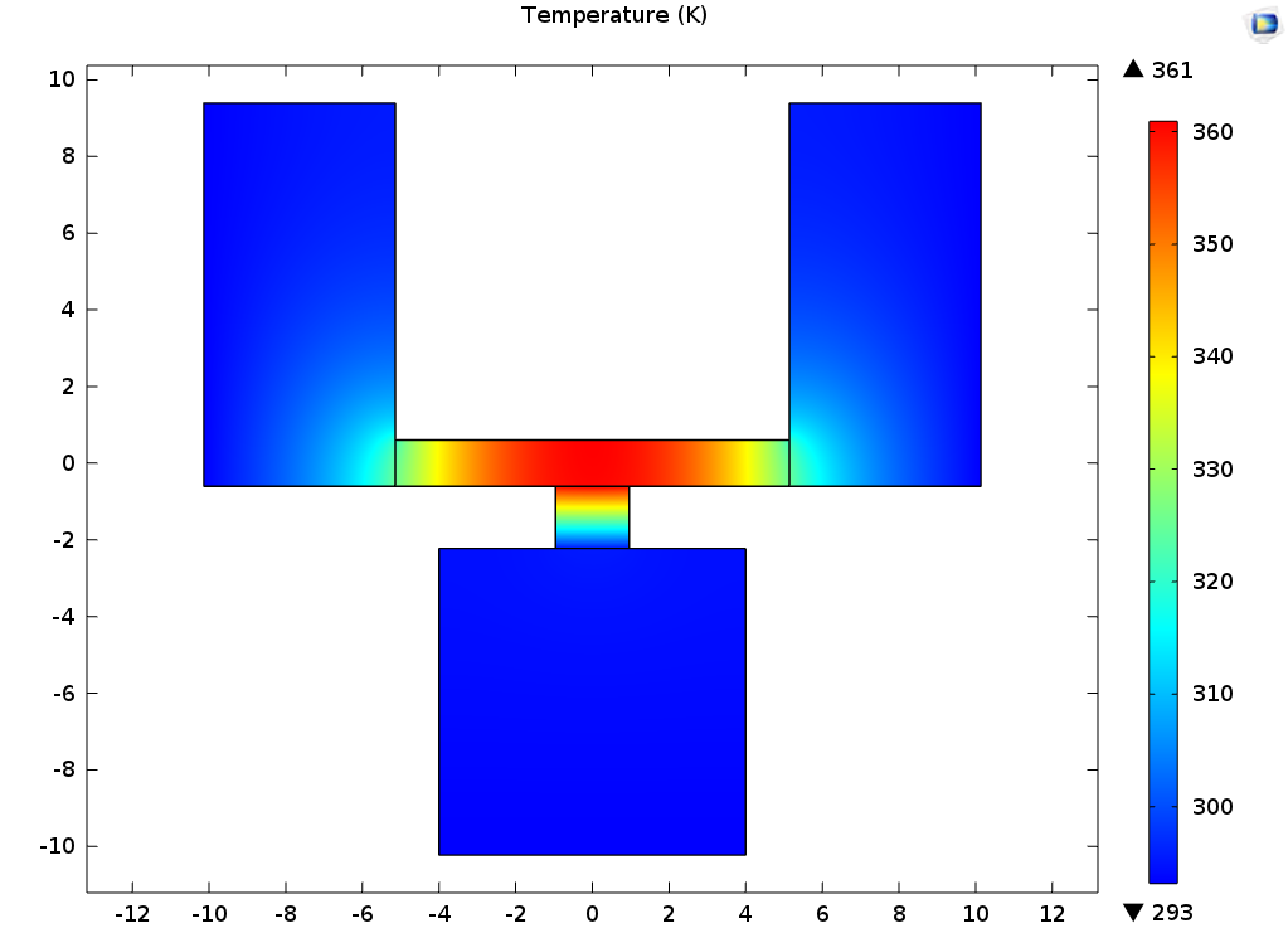


**Figure S9 Temperature distribution of Au film sensor with and without graphene.**

Figure S9 gives one example of thermal analysis in SLG5. It is clear to see that as the temperature at the end of sensor increases, both temperatures of bare sensor and T-type sensor increase, while their temperature difference does not change significantly. In the T-type sensor method, the thermal conductivity of graphene only depends on the temperature difference of Au film sensor with and without graphene. The accuracy of finite-element model depends on the correct Joule heating power, dimensions and thermal properties of Au film, wherein the largest error comes from the sensor’s thermal conductivity, about 5%. We changed the input thermal conductivity of sensor by 5% and found that the thermal conductivity of graphene was changed by 5.8%. Including the other factors, the final uncertainty of thermal conductivity is estimated to be about 10%.

It is noted that a relatively large temperature gradient was applied to the graphene sample in the experiment. The reason is that the purpose of this work is to understand how the different kinds of defects affect the thermal conductivity of graphene. Before and after defect-engineering on graphene, the detectable temperature change of Au film sensor is around 1 K for 50 K temperature gradient. If small temperature gradient around 10 K is used, it is very difficult to directly evaluate the influence of defect. On the other hand, if we only wish to measure the thermal conductivity of graphene without any defect-engineering, smaller temperature gradient is enough. We could decrease the temperature gradient to about 17 K to obtain 1 K detectable temperature change.

It is well known that the thermal conductivity of graphene is temperature-dependent. Unfortunately, the possible temperature range of our constant-temperature stage is only from -10 ˚C to 40 ˚C. In this work, the temperature of graphene is decided as the average value based on the finite-element calculation. The temperature of Au film sensor was measured precisely through its resistance change. Four-wire method and high-precision digital multimeter guaranteed 0.1 K temperature resolution. The temperature rise at the end of sensor was calculated by a finite-element method. The input thermal conductivity of sensor and Joule heating power were measured in the experiment. The uncertainty of sensor’s thermal conductivity was 5%, leading to 4.6% temperature uncertainty at the end of sensor. As a result, the average temperature rise of graphene can be decided within 5% uncertainty.

**6. Contact thermal resistance between graphene and metal film sensor**

In our experiment, the Au film was directly deposited on graphene. Before etching SiO_2_, the target graphene ribbon and 100 nm thick SiO_2_ layer were suspended above the silicon substrate. The etching rate of SiO_2_ under the suspended graphene was two times as large as that of SiO_2_ layer covered by the Au film. We carefully controlled the wet etching time not to completely remove the SiO_2_ under the Au film.


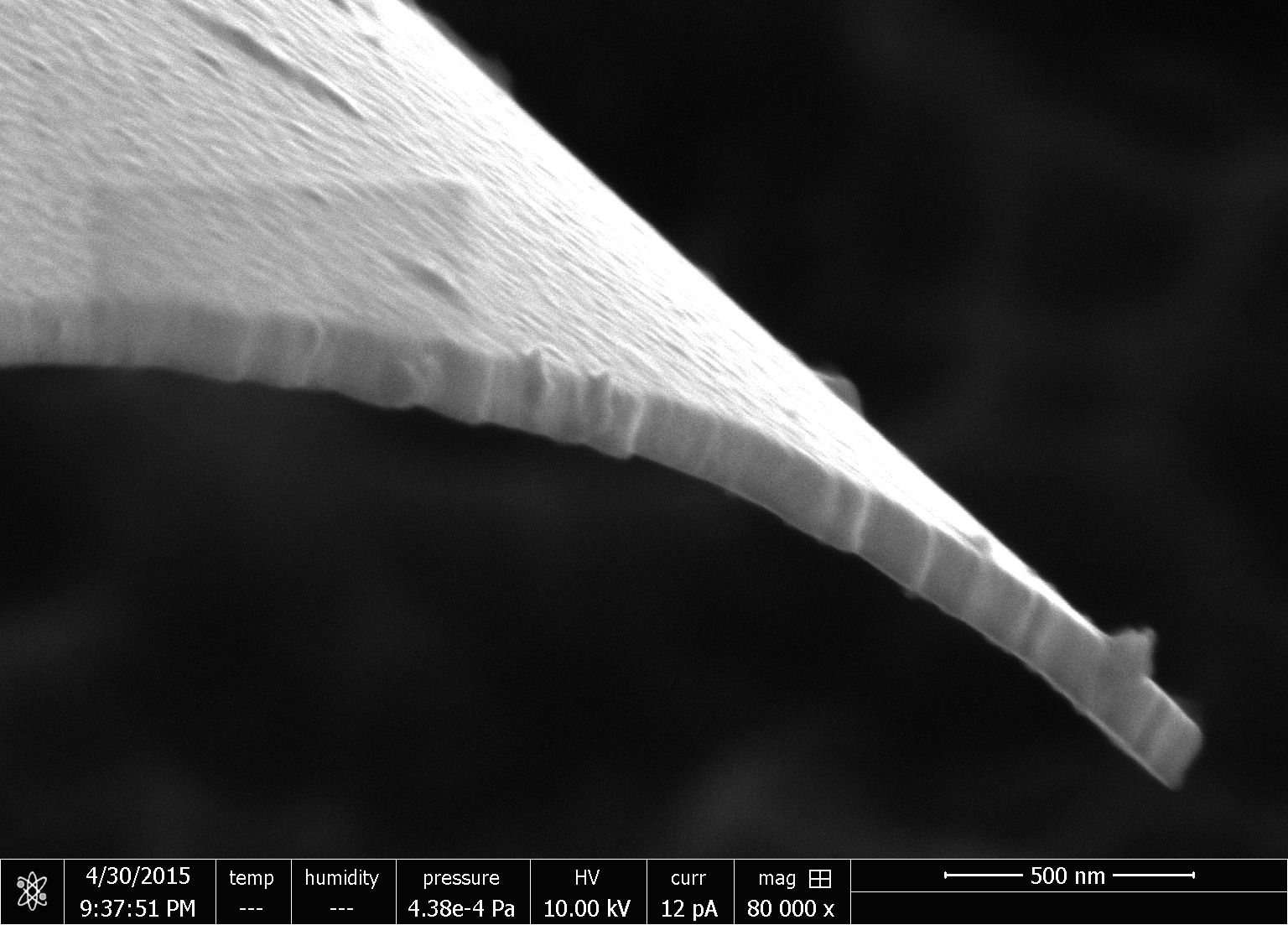

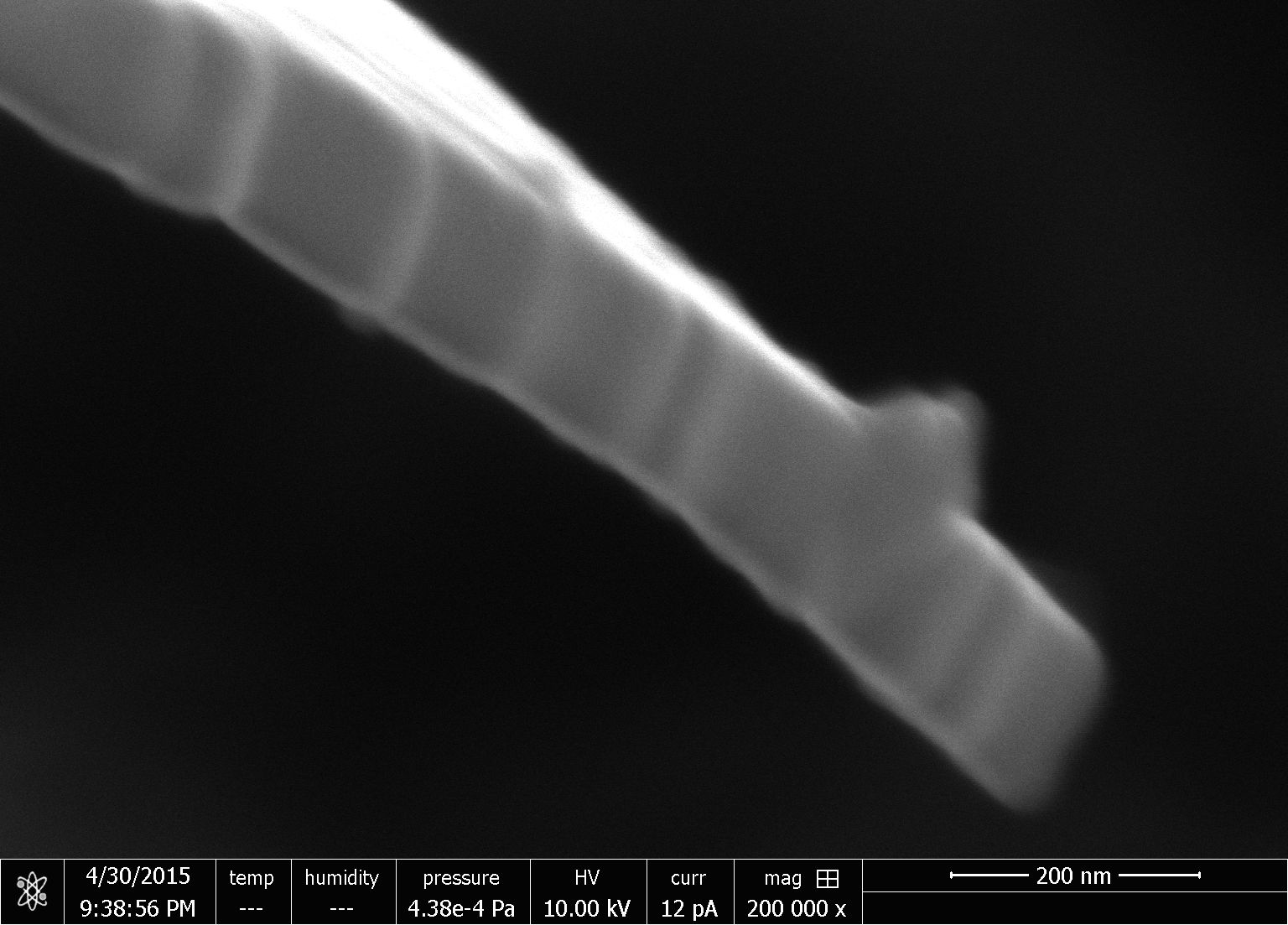


**Figure S10 Suspended Au film after SiO_2_ wet etching process.**

Figure S10 shows the SEM image of suspended Au film after etching SiO_2_ layer beneath. The thickness of film is measured to be 150 nm, wherein the thickness of metal film is 10 nm Cr layer and 100 nm Au layer, measured by a quartz crystal film thickness monitor. Thus, the thickness of SiO_2_ layer is 40 nm. The graphene segment was clamped between the Au film sensor and SiO_2_ layer. This sandwich structure will help to improve the heat contact between the graphene and Au film sensor. As for the other suspended graphene samples in literature, the graphene is usually transferred onto the thin film electrode. Because small amount of air or impurities may be trapped between the graphene and metal film during the transfer process, sometimes the electron beam induced deposition is needed to create metallic micro-ribbon on the graphene to improve thermal contact.

We calculated the contact thermal resistances of all the graphene samples. The results are given in Table 1. In this table, *L*, *W*, *L_c_* are the graphene ribbon length, width and contact length between the graphene and gold thin film sensor. *λ*_0_ is the thermal conductivity of graphene calculated from the finite element heat conduction analysis without considering the contact thermal resistance. *R*_0_ is the total thermal resistance. *R_fin_* is the contact thermal resistance calculated by a fin model^1^. *R_sim_* is the thermal resistance calculated by a simple formula *R_c_* = *R_int_* /*S*, where *R_int_* = 4×10^-8^ m^2^K/W is the interfacial thermal resistance per unit area and *S* is the contact area.

Table 1. Contact thermal resistances of all graphene samples

|  | *L*  (μm) | *W*  (μm) | *L_c_*  (μm) | *λ*_0_  (Wm^-1^K^-1^) | *R*_0_×10^6^  (KW^-1^) | *R_fin_*×10^5^  (KW^-1^) | *R_sim_*×10^4^  (KW^-1^) | *R_fin_/R*_0_  ( - ) | *R_sim_/R*_0_  ( - ) |
| --- | --- | --- | --- | --- | --- | --- | --- | --- | --- |
| SLG#1 | 1.86 | 0.42 | 1.13 | 1287 | 10.12 | 7.20 | 8.41 | 0.071 | 0.008 |
| SLG#2 | 1.56 | 0.85 | 1.29 | 2177 | 2.48 | 2.74 | 3.66 | 0.110 | 0.015 |
| SLG#3 | 1.65 | 1.22 | 1.18 | 2331 | 1.71 | 1.84 | 2.78 | 0.108 | 0.016 |
| SLG#4 | 1.62 | 1.30 | 1.21 | 1783 | 2.06 | 1.98 | 2.55 | 0.096 | 0.012 |
| SLG#5 | 1.62 | 1.92 | 1.16 | 2600 | 0.95 | 1.11 | 1.79 | 0.116 | 0.019 |
| SLG#6 | 1.73 | 2.08 | 1.12 | 2554 | 0.96 | 1.03 | 1.71 | 0.108 | 0.018 |
| SLG#5 1s FIB | 1.62 | 1.32 | 1.12 | 1690 | 2.14 | 2.00 | 2.70 | 0.094 | 0.013 |

1. (SLG #5 1s FIB) is the sample SLG#5 radiated by FIB for 1s.

2. *λ*_0_ is the value of graphene with an average temperature around 310 K.

As summarized in Table 1, the contact thermal resistance predicted by the fin model contributes about 10% to the total thermal resistance of graphene. This ratio is higher for the sample with higher thermal conductivity. Meanwhile, the contact thermal resistance calculated by the simple formula *R_c_* = *R_int_* /*S* is less than 2% of the total thermal resistance. The difference between these two models depends on the temperature change of graphene segment in contact with the metal film along its length direction. If the thermal contact resistance between the graphene and metal film is small enough, the temperature of graphene segment supported by metal film tends to be uniform and the simple formula can be well applied. From this point of view, the fin model gives a high limit to the contact thermal resistance.

**7. Parameter analysis of the theoretical model**

Based on the theory of phonon dynamics with full phonon dispersion relation, the thermal conductivity of suspended SLG is calculated to compare with the experimental data and analyze the underlying mechanisms. In the present model, several parameters are important for determining the thermal conductivity, such as: phonon dispersion relation, group velocity, Gruneisen parameter, size of SLG, cut-off frequency of each phonon branch, specularity parameter at boundary, etc. Here, we chose three most important parameters for analysis.


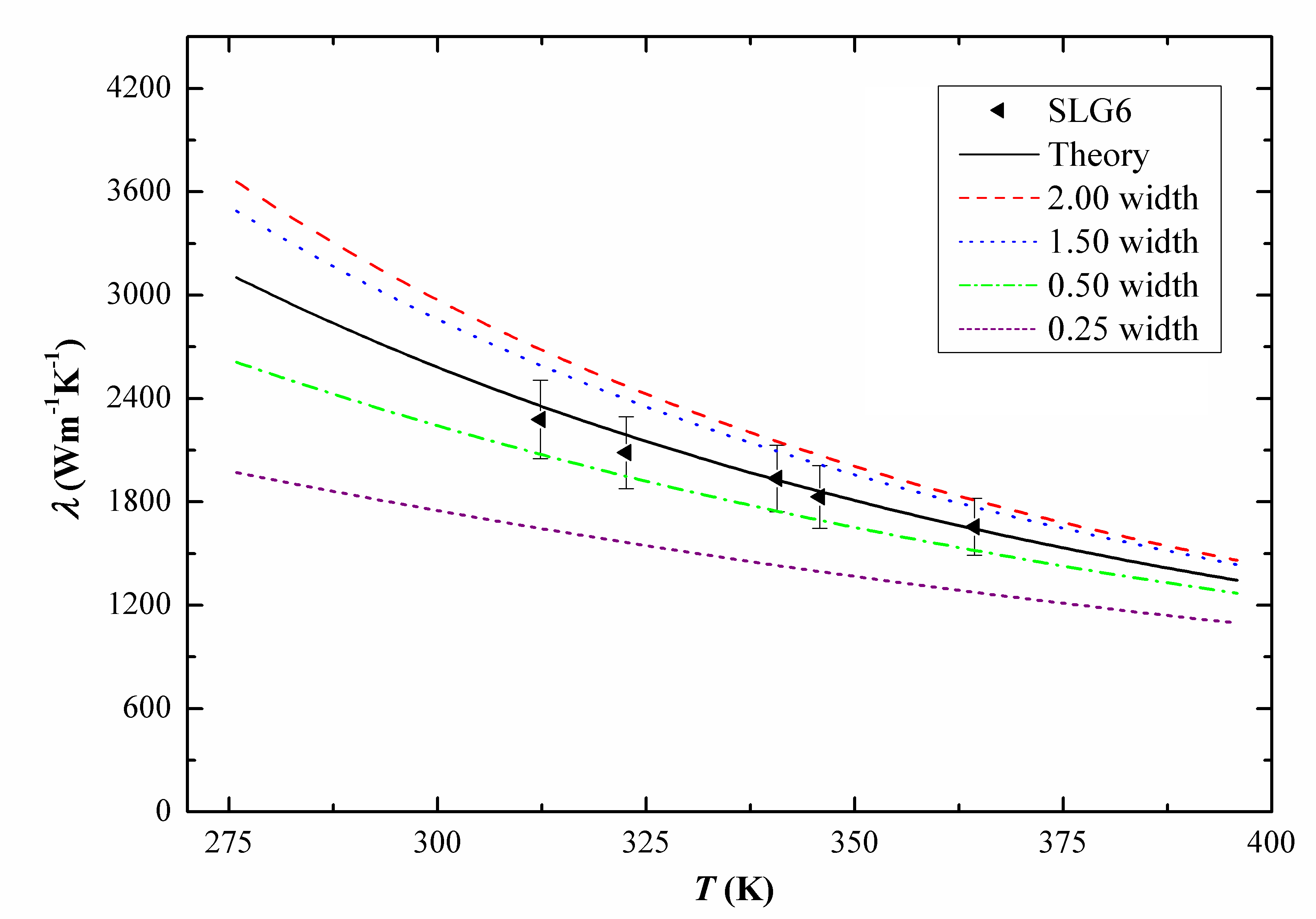


**Figure S11 Thermal conductivity of SLG calculated with different widths.**

The experimental data of SLG6 is used as a basis for the parameter analysis. Fig. S11 shows the calculated thermal conductivity changed with different widths of SLG. The other parameters are kept unchanged. It is seen that *λ* increases as the width increases and the increment is more significant at low temperatures. On the other hand, the increasing *λ* with increasing SLG width has a convergence value.


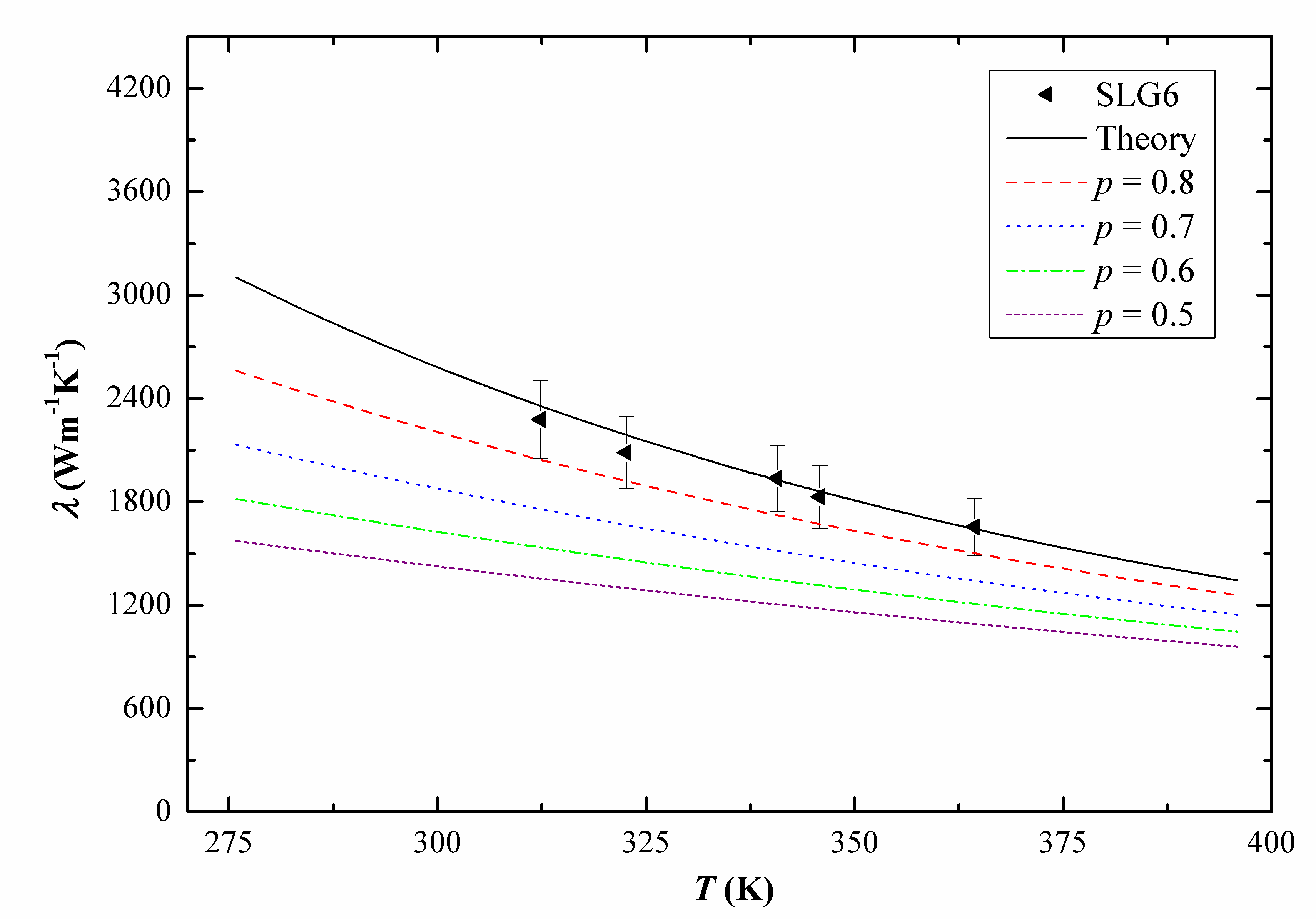


**Figure S12 Thermal conductivity of SLG calculated with different specularity parameter *p*.**

Figure S12 shows the calculated thermal conductivity changed with different specularity parameter *p*. For the sample SLG6, *p* equals 0.9. As the parameter *p* decreases, more phonons are scattered at the graphene boundary, i.e., the edge of graphene is rougher and the thermal conductivity decreases. In our experiment, the sample SLG1 has the lowest *p* because of the obvious edge folding effect.


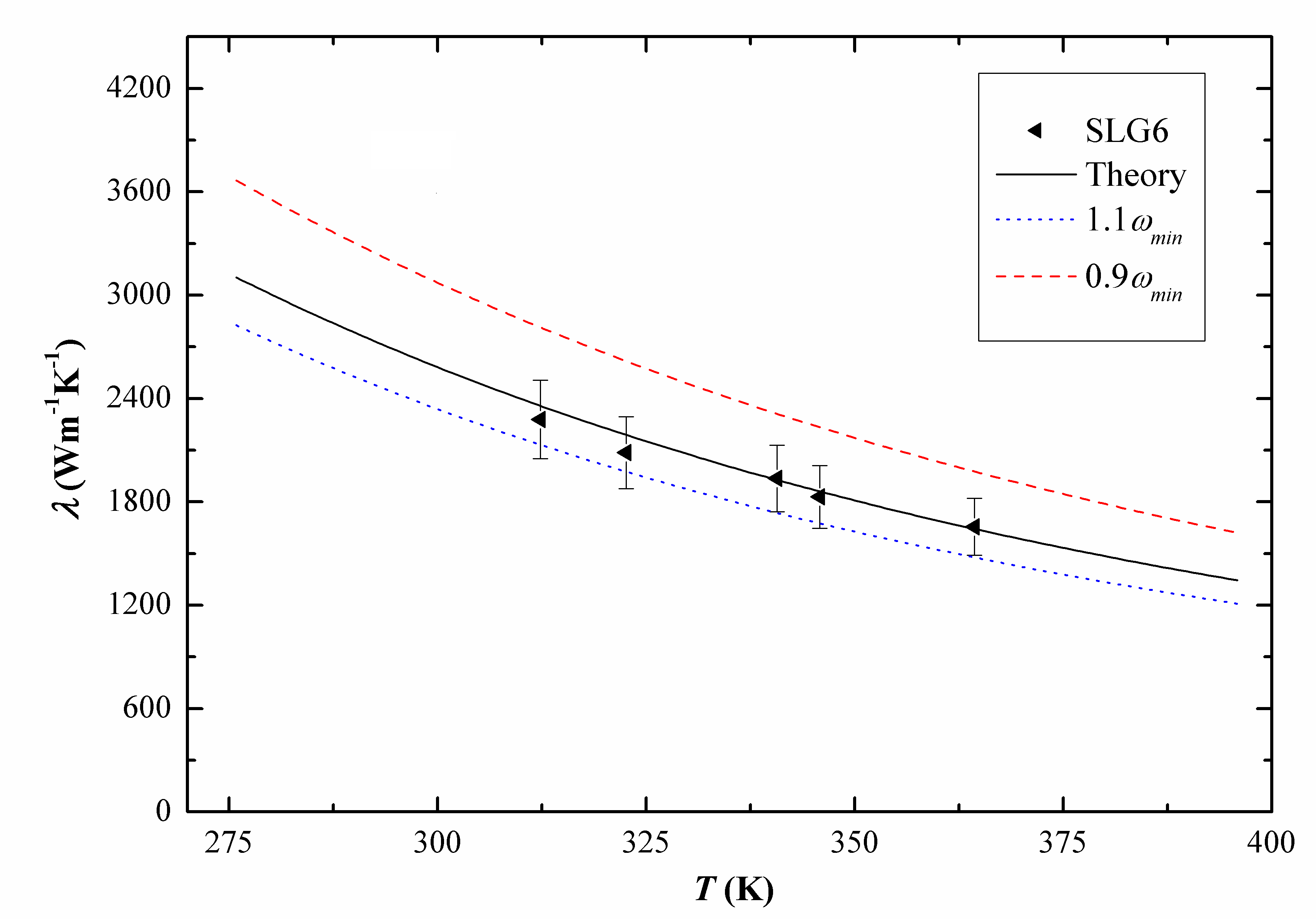


**Figure S13 Thermal conductivity of SLG calculated with different minimum cut-off frequency.**

Another important parameter for deciding *λ* is the minimum cut-off frequency *ω_min_*. *ω_min_* corresponds to the phonons in the center of the first Brillouin zone of SLG, where phonons have large group velocity and long life time. Phonons of LA and TA modes are dominant for heat conduction in SLG. *λ* is sensitive to the *ω_min_* of these two modes. Fig. S9 shows the thermal conductivity of SLG with *ω_min_* changed by ±10%. We found that *ω_min_* is affected by the defect level and the temperature of SLG. Meanwhile, *ω_min_* of SLG is much smaller than the bulk graphite, corresponding to the 2D nature of SLG.

**References**

1. Pettes M. T., Jo I., Yao Z. & Shi L. Influence of polymeric residue on the thermal conductivity of suspended bilayer graphene. *Nano Lett*. 11, 1195-1200 (2011).
2. Cancado, L. G. et al. Quantifying defects in graphene via raman spectroscopy at different excitation energies, *Nano Lett.* **11**, 3190 (2011).
3. Shivaraman, S. et al. Free-standing epitaxial graphene, *Nano Lett.* **9**, 3100 (2009).
4. Jang, W., Bao, W., Jing, L., Lau, C. N. & Dames, C. Thermal conductivity of suspended few-layer graphene by a modified T-bridge method. *Appl. Phys. Lett.* **103**, 133102 (2013).
5. Fujii M., Zhang X., Xie H. Q., Ago H., Takahashi K., Ikuta T., Abe H. & Shimizu T. Measuring the thermal conductivity of a single carbon nanotube. *Phys. Rev. Lett.* 95, 065502 (2005).
